# Problematic Mobile Phone Use and Addiction Across Generations: the Roles of Psychopathological Symptoms and Smartphone Use

**DOI:** 10.1007/s41347-017-0041-3

**Published:** 2018-01-08

**Authors:** Daria J. Kuss, Eiman Kanjo, Mark Crook-Rumsey, Fraenze Kibowski, Grace Y. Wang, Alex Sumich

**Affiliations:** 1 Nottingham Trent University, 50 Shakespeare Street, Nottingham, NG1 4FQ UK; 2 Auckland University of Technology, North Shore Campus Northcote, Auckland, 1142 New Zealand

**Keywords:** Problematic mobile phone use, Smartphone addiction, Psychopathology, Stress, Depression, Anxiety, Generation X, Generation Y

## Abstract

Contemporary technological advances have led to a significant increase in using mobile technologies. Recent research has pointed to potential problems as a consequence of mobile overuse, including addiction, financial problems, dangerous use (i.e. whilst driving) and prohibited use (i.e. use in forbidden areas). The aim of this study is to extend previous findings regarding the predictive power of psychopathological symptoms (depression, anxiety and stress), mobile phone use (i.e. calls, SMS, time spent on the phone, as well as the engagement in specific smartphone activities) across Generations X and Y on problematic mobile phone use in a sample of 273 adults. Findings revealed prohibited use and dependence were predicted by calls/day, time on the phone and using social media. Only for dependent mobile phone use (rather than prohibited), stress appeared as significant. Using social media and anxiety significantly predicted belonging to Generation Y, with calls per day predicted belonging to Generation X. This finding suggests Generation Y are more likely to use asynchronous social media-based communication, whereas Generation X engage more in synchronous communication. The findings have implications for prevention and awareness-raising efforts of possibly problematic mobile phone use for educators, parents and individuals, particularly including dependence and prohibited use.

## Introduction

Recent technological advances have led to a significant increase in using mobile technologies. The communications regulator Ofcom (2016) refers to the UK as “smartphone society”: 93% of the population own a smartphone, and users spend more time online on their phone (approximately 20 h/week) than using other devices (e.g. laptops and desktop-computers). These recent trends suggest mobiles and the Internet have become intimately intertwined to enable “on-the-go” access to several facilities (i.e. web-browsing, communication, shopping, banking and gaming) (Ofcom 2016).

With the ubiquity and convenience of using mobiles for Internet access, recent research suggests that problematic mobile phone use may consist of several factors, including addiction/dependence (Billieux et al. 2008; Chóliz 2010), financial problems (Billieux et al. 2008), dangerous use (i.e. whilst driving) (Bianchi and Phillips 2005; White et al. 2004) and prohibited use (i.e. in forbidden areas) (Nickerson et al. 2008). From an epidemiological perspective, prevalence rate estimates vary considerably, and there is limited knowledge and understanding about the aetiology of problematic and addictive smartphone use, including its course (Billieux et al. 2015). Nonetheless, the World Health Organization (2015) considers addictive mobile phone use as public health concern, emphasising the need for more research concerning risk factors and course specifiers.

Using mobile Internet may increase habitual checking behaviours, which may contribute to developing psychopathological symptoms, such as addiction symptoms (Jeong et al 2016). The recent addition of a behavioural addiction category in the most recent edition of the diagnostic manual (DSM-5) (American Psychiatric Association 2013) including *Gambling Disorder* as first official diagnosis in that category and *Internet Gaming Disorder* as condition requiring further research to be included in the main manual (Kuss et al. 2016) suggests other potential behavioural addictions, e.g. mobile phone addiction, may be included in the manual if they lead to clinically significant impairment. Accordingly, using a biopsychosocial model of addiction, mobile phone or smartphone dependence may be considered to fall within the spectrum of behavioural addictions, including symptoms such as salience, mood modification, withdrawal, tolerance, conflict and relapse (Griffiths 2005), if it poses a significant (mental) health concern for the affected individual. Young individuals specifically appear as enthusiastic adopters of mobile technologies, and research suggests teenagers and young adults are considered particularly at risk for developing mobile phone addiction (Jeong et al. 2016; Kwon et al. 2013).

Generation X (born until the early 1980s) and Generation Y (younger individuals, born in the mid-80s and later) have been found to differ in terms of their adoption of smartphone technology (Gafni and Geri 2013), and research suggests that younger individuals may be more likely to develop problems as a consequence of their excessive use of new technologies (Echeburua and de Corral 2010). Research using Swiss vocational school students furthermore suggests that smartphone addiction is more prevalent in younger adolescents in comparison to adults (Haug et al. 2015), indicating that younger individuals may be particularly at risk for developing problematic mobile phone use. From a developmental perspective, psychosocial maturation may contribute to developing more resilience to stressors in the process of maturation (Motti-Stefanidi 2015). As individuals grow older, this may decrease the risk for psychopathological symptom experience and possibly associated maladaptive coping in the form of increased mobile phone use. Consequently, it can be hypothesised that both problematic mobile phone use and psychopathological symptoms experience will be more pronounced in younger age groups, i.e. Generation Y, in comparison to Generation X.

Previous research (Ha et al. 2008; Hong et al. 2012; Panova and Lleras 2016) found excessive use of mobile phones was related to anxiety, depression and general distress. Research has suggested individuals with social anxiety symptoms prefer using mobile phones for text-based asynchronous communication as this may alleviate fears associated with face-to-face and synchronous interactions (Park et al. 2010; Reid and Reid 2007), potentially contributing to problematic use. It has also been suggested the preference for text-based asynchronous communication (which is often favoured by Generation Y) may exacerbate anxious social behaviours, as physical cues vital for social interaction are not present during these interactions, and the possibilities of developing social confidence and skills are consequently limited (Panova and Lleras 2016).

Depression was also linked to dependent mobile phone use in another study using young university students in Iran (Babadi-Akashe et al. 2014). Individuals who feel depressed may use mobile phones to seek social support and cope with their loneliness and apathy feelings, which may exacerbate feelings of depression and stress (Murdock 2013). Further research (Jeong et al. 2016) also highlighted that stress predicts smartphone addiction. As enthusiastic adopters of new technology, Generation Y may use their smartphones to cope with everyday stressors (e.g. social situations and relationship problems), and using the phone as coping mechanism can be considered dysfunctional, similar to using the Internet to cope with life problems (Kuss et al. 2017), potentially leading to symptoms traditionally associated with substance-related addictions (Kuss et al. 2014).

Previous research on excessive Internet use with young individuals, i.e. both adolescent (Haug et al. 2015; Kuss et al. 2013b) and university student samples (Kuss et al. 2013a), has indicated technology use is not necessarily problematic per se; however, the use of particular online applications, e.g. gaming (Kuss 2013) and social networking (Haug et al. 2015; Kuss and Griffiths 2017), may put young individuals at risk for developing addiction-related problems (Jeong et al. 2016). Similar findings have been produced by a recent cross-cultural study (Lopez-Fernandez et al. 2017) involving Generation Y participants from ten European countries, suggesting gaming and social networking may put individuals at risk for developing addiction-related problems through excessive mobile phone use. Another study using elementary school children in South Korea (Jeong et al. 2016) found although both social networking and gaming on smartphones may increase the risk of smartphone addiction, using social networking sites was a stronger predictor than gaming.

Moreover, research (Lopez-Fernandez et al. 2017) using young individuals highlighted further potential risk factors for mobile phone addiction, namely female gender and time spent using mobile phones. In general, females appear to place stronger emphasis on social interactions than males which may make them more prone to developing problems due to increased mobile phone use. Similarly, time spent using mobile phones and engaging in mobile phone-related activities (e.g. sending text messages and making calls) may contribute to habitualisation effects, including frequent checking (Kanjo et al. 2017), which may increase the consequential experience of addiction-related problems.

The aim of this study is to replicate and add to previous findings and fill the gaps in knowledge regarding indicators and predictors of problematic mobile phone use. Based on previous research, it is hypothesised female gender, depression, anxiety, stress and specific mobile phone usage (i.e. time spent, texts sent, calls made, using social networking and gaming, respectively) significantly predict problematic mobile phone use, with these effects mediated by age group (i.e. Generation X vs Generation Y), so they are more pronounced in Generation Y. This study specifically aims to differentiate between different types of problematic mobile phone use, namely dependent, dangerous/prohibited use and financial problems through mobile phone use, as these have been pointed out as viable constructs in previous research (Billieux et al. 2008; Nickerson et al. 2008; White et al. 2004).

## Method

### Participants

Participants (*N* = 273; aged 16–65 years [*M* = 28.31, *SD* = 11.1; 74% females]) from 14 different countries (74.36% UK, 18.32% New Zealand) were recruited through Internet advertising on Android-phone forums and university participant-recruitment pools. A binary age variable was created for comparison between Generation X (31–65 years, 59 women and 24 men, coded as 1) and Generation Y (16–30 years, 143 women and 45 men, coded as 0). There were no significant sex differences between the generations (chi-square = 0.752 (1), *p* = 0.45).

### Measures

#### Problematic Mobile Phone Use

The 30-item Problematic Mobile Phone Use questionnaire (PMPU) (Billieux et al. 2008) uses 4-point Likert-type scales which measure four distinct domains dimensionally, with higher scores indicating more problematic use: (i) dangerous use, defined as using a mobile phone in dangerous situations (e.g. whilst driving, 5 items); (ii) prohibited use, i.e. using mobile phones if forbidden to do so (e.g. in the library, 5 items); (iii) dependence, i.e. presence of addictive symptoms (e.g. feelings of loss without the phone, 7 items) and (iv) financial problems, i.e. monetary problems as direct result of phone use (e.g. receiving high mobile phone bills, 13 items). A 4-factor solution showed good fit to the data in confirmatory factor analyses (Billieux et al. 2008), and the PMPU’s external and internal validity have been validated (Billieux 2012). Cronbach’s alphas have demonstrated excellent levels of reliability for the dependence (*α* = .85) and financial problems (*α* = .89) subscales and acceptable levels for the prohibited use (*α* = .67) and dangerous use subscales (*α* = .74) (Billieux et al. 2008).

#### Depression, Anxiety and Stress

The 42-item DASS-42 was used to measure depression, anxiety and stress (14 items each) by means of 4-point Likert-scales (Crawford and Henry 2003). The depression subscale measures hopelessness, devaluation of life, dysphoria, self-depreciation, lack of interest or involvement, inertia and anhedonia. The anxiety subscale assesses anxious effects, situational anxiety, subjective experience, autonomic arousal and skeletal muscle effects. The stress scale measures overreaction, impatience, difficulty relaxing, nervous arousal and being upset easily. High levels of convergent validity have been demonstrated with the depression subscale of the Personal Disturbance Scale (.78) (Bedford and Foulds 1978) and the DASS depression subscale correlated with the depression scale of the Hospital Anxiety and Depression Scale (.66) (Zigmond and Snaith 1983). Excellent levels of reliability have been demonstrated for the depression, anxiety and stress scales (*α* = .91, .84 and .90), respectively (Lovibond and Lovibond 1995).

#### Phone Use

Phone use was measured by inquiring about calls, SMS, time spent on the phone, as well as the engagement in specific smartphone activities. Accordingly, participants were asked the following questions regarding their phone use:

“How many calls do you make with your mobile phone per day?” was assessed using multiple choice answers: “0–2”, “3–5” or “More than 5”. “How many SMS (text messages) do you send per day?” was assessed using multiple choice answers: “0–3”, “4–10” or “More than 10”. “How much time do you spend on your mobile phone per day?” was answered as follows: “0–10 min”, “10–30 min” or “More than 30 min”. “On average, how many times per day do you use your phone for Internet browsing, social media, and games?” was ranked on a 7-point Likert-scale.

### Statistical Analyses

The analyses were based on previously used mediator models (Sitko et al. 2014). The models were specified using Mplus version 8 (Muthén and Muthén 2011) and estimated using the maximum likelihood estimator for the first part and weighted least squares estimation for the second part (due to the binary mediator). For all analyses, STDYX standardised estimates are being reported.

**Fig. 1:**
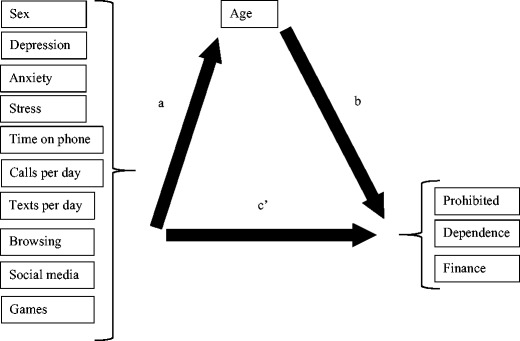
The mediator model

The first part of the two-part analysis estimated the direct effects (c-paths) between the predictor variables (sex, depression, stress, anxiety, calls/day, time on phone, texts/day, ranked phone use for Internet browsing, social media and games) and outcome variables (PMPU subscales) without the mediator. The second part introduced the binary mediating variable age (see Fig. 1). The model now included the direct effects between the predictor variables and the mediator (a-paths), the direct effects between the mediator and the outcome variables (b-paths), and the direct effects between predictor and outcome variables, while controlling for the mediator (c’-paths). The meditated effects are the products of the *a*- and b-paths. There are two contexts for the term mediating effect. One, a previously significant c-path becomes non-significant with the inclusion of the mediator (*c*’ = 0), and the mediating effect is significant, suggesting the relationship between the predictor and outcome variable is fully mediated by age. Two, both the c- and c’-paths are significant with at least one significant mediating effect, suggesting a partial mediation. When a previously non-significant *c*-path remains non-significant (*c*’ = 0), but the mediating variable is significantly related to at least one predictor and one outcome variable, the term indirect effect is used, indicating any relationship between predictor and outcome is entirely contingent on the mediator.

## Results

Mean scores for the DASS42 were 9.16 for depression (*SD* = 8.74, range 0–41), 6.87 for anxiety (*SD* = 6.69, 0–31) and 13.29 for stress (*SD* = 9.32, 0–37); for the PMPU subscales: 9.69 for dangerous (*SD* = 2.08, 8–17), 9.71 for prohibited (*SD* = 3.06, 5–19), 18.56 for dependence (*SD* = 5.47, 7–28) and 23.11 for financial (*SD* = 5.63, 13–46). All scales, apart from the DASS42 depression and anxiety subscales and the PMPU financial subscale, satisfied measures of skew and kurtosis. All scales displayed good Cronbach’s alpha levels above 0.70, other than the PMPU Prohibited (0.63 adequate) and dangerous subscales (0.422 poor). Descriptive statistics and Cronbach’s alpha values are displayed in Table 1. Due to its low alpha score, the dangerous subscale was not included in the analysis. There was no multi-collinearity present in the predictor variables. The highest correlation was observed between the stress and anxiety subscale at 0.78 (*p* < 0.001).

**Table 1:** Descriptive statistics and Cronbach’s alpha values

	Mean	SD	Range	Skew	Kurtosis	Cronbach’s alpha	Missing
DASS42							
Depression	9.16	9.12	0–41	1.507	2.053	0.942	11
Anxiety	7.03	6.90	0–40	1.574	2.961	0.887	12
Stress	13.39	9.63	0–41	0.723	−0.139	0.936	8
PMPU							
Dangerous	9.69	2.08	8–17	1.441	1.934	0.42	11
Prohibited	9.71	3.06	5–19	.499	−.052	0.63	3
Dependence	18.56	5.47	7–28	−.076	−.923	0.843	2
Financial	23.11	5.63	13–46	1.429	3.090	0.824	14

Regarding the additional phone use questions, 71.4% participants reported making 0–2 calls per day, 22% reported 3–5 calls per day and 6.2% making more than 5 calls per day; the remaining 0.4% of data were missing. Texts per day were more evenly distributed with 34.9% reporting 0–3 texts per day, 30.7% reported 4–10 and 33.2% reported more than 10 per day, with 1.4% missing data. Of the participants, 4% reported 0–10 min in terms of time on phone per day, 7.7% reported 10–30 min and 86.9% reported more than 30 min per day, with 1.5% missing data. The mean for ranking Internet browsing was 5.15 (*SD* = 1.75), social media 5.37 (*SD* = 1.81) and games 2.62 (*SD* = 1.90) with skew and kurtosis within acceptable limits (+/−2). Moreover, calls per day seemed to have a flooring effect, suggesting most people tended to use their phones for fewer calls, whilst time on the phone was found to have a ceiling effect, which suggests that people spent more than 30 min on their phones per day on average. Due to missing data on categorical predictor variables, 18 participants were excluded from the analyses (*N* = 255).

### Direct Effects

For part one of the analysis (*N* = 255, ML estimator), the direct effects (c-paths) for prohibited and dependence were similar, with significant paths for calls per day, time on phone and social media. In addition, stress showed a significant, positive relationship with dependence only. For finance, only social media displayed a significant, positive relationship. All c-paths are reported in Table 2. For the second part of the analyses (*N* = 231, WLSM estimator), the significant direct effects (a-paths) of the predictors on the binary age variable anxiety and social media showed a negative relationship. Calls per day displayed a positive relationship with age; results are reported in Table 3. Regarding the direct effects (b-path) of age on the PMPUQ subscales, only prohibited use showed a negative relationship with age, reported in Table 4.

**Table 2:** Results of direct effects (c-paths) between predictors and outcome variables

Predictors	Prohibited B (SE)	Dependence B (SE)	Finance B (SE)
Sex (female)	− 0.063 (0.059)	0.028 (0.059)	0.051 (0.064)
Depression	0.023 (0.081)	− 0.063 (0.081)	0.011 (0.091)
Anxiety	0.042 (0.097)	− 0.063 (0.100)	0.190 (0.107)
Stress	0.139 (0.100)	0.329 (0.101)**	− 0.068 (0.112)
Time on phone	0.216 (0.058)***	0.152 (0.059)*	− 0.022 (0.067)
Calls per day	0.170 (0.059)**	0.160 (0.059)**	0.105 (0.064)
Texts per day	0.065 (0.059)	0.084 (0.059)	0.077 (0.064)
Browsing	− 0.054 (0.064)	− 0.030 (0.064)	− 0.058 (0.071)
Social media	0.252 (0.065)***	0.229 (0.066)**	0.160 (0.073)*
Games	0.008 (0.057)	0.020 (0.058)	− 0.019 (0.063)

*B* standardized b coefficients, *SE* standard error

*p < 0.05; **p < 0.01; ***p < 0.001

**Table 3:** Results of direct effects (a-paths) between predictors and mediator

Predictors	Age *B (SE)*
Sex	− 0.008 (0.086)
Depression	− 0.061 (0.106)
Anxiety	− 0.499 (0.139)***
Stress	0.196 (0.152)
Time on phone	− 0.111 (0.075)
Calls per day	0.189 (0.087)*
Texts per day	− 0.127 (0.101)
Browsing	0.140 (0.105)
Social media	− 0.217 (0.094)*
Games	0.040 (0.081)

*B* standardized b coefficients, *SE* standard error

*p < 0.05; **p < 0.01; ***p < 0.001

**Table 4:** Results of direct effects (b-paths) between mediator and outcome variables

Mediator	Prohibited B (SE)	Dependence B (SE)	Finance B (SE)
Age (31–65 years)	− 0.150 (0.071)*	− 0.070 (0.088)	− 0.127 (0.101)

*B* standardized b coefficients, *SE* standard error

*p < 0.05; **p < 0.01; ***p < 0.001

Comparable to the c-paths, similar trends in significant predictors were seen for the c’-paths for dependence, with stress, calls per day, time on phone and social media remaining significantly and directly related to dependence when age was controlled for and included as a mediator (c’-path Table 5). For finance, the significant c-path of the first part of the analysis (Table 2) is no longer significant once age was controlled for and included as a mediator (c’-path Table 5). For prohibited use, the direct relationship of time on the phone (c-path) remained significant once age was controlled for (c’-path Table 5).

**Table 5:** Results of direct effects (c’-paths) between predictors and outcome variable including the binary mediator age

Predictors	Prohibited B (SE)	Dependence B (SE)	Finance B (SE)
Sex (female)	− 0.068 (0.067)	0.020 (0.065)	0.030 (0.067)
Depression	− 0.004 (0.068)	− 0.086 (0.079)	0.006 (0.110)
Anxiety	− 0.026 (0.107)	− 0.095 (0.110)	0.105 (0.118)
Stress	0.169 (0.111)	0.380 (0.106)***	− 0.006 (0.130)
Time on phone	0.179 (0.079)*	0.137 (0.057)*	− 0.058(0.054)
Calls per day	0.223 (0.061)***	0.185 (0.066)**	*0.145 (0.074)*
Texts per day	0.030 (0.063)	0.051 (0.062)	0.068 (0.069)
Browsing	− 0.024 (0.080)	0.015 (0.068)	0.003 (0.084)
Social media	0.178 (0.077)*	0.149 (0.072)*	0.087 (0.083)
Games	0.024 (0.066)	0.008 (0.070)	− 0.047 (0.073)

*B* standardized b coefficients, *SE* standard error

*p < 0.05; **p < 0.01; ***p < 0.001

Result in italics *p* = 0.05

### Mediating and Indirect Effects (c’, See Table 5)

When the effect of the mediator was included in the model, age partially mediated the relationship between calls per day and using social media and prohibited phone use. Calling often in a day related directly to a higher score on prohibited use, but it also made it more likely to belong to the older age category (Generation X) compared to the younger one (Generation Y), which in turn was related to a lower score on prohibited use. Therefore, while calling often directly increased the outcome variable prohibited use, there was a simultaneous muting of that effect through the mediator age. This gives a more detailed and differentiated view on the findings of part one of the analyses, in which calling often per day was related to a higher score in prohibited use (c-path). Ranking social media highly was related directly to higher scores on prohibited use, but also to belonging to the younger age category (Generation Y) compared to the older one (Generation X), which in turn also related to a higher prohibited use score.

There was an indirect relationship between anxiety and prohibited use via the mediator age. While anxiety had no direct relationship to prohibited use in part one of the analysis (c-path) and no direct relationship to prohibited use in part two (c’-path), it was related to belonging to the younger age category (Generation Y) compared to the older (Generation X), which in turn related to higher prohibited use. The total proportion of variance that was significantly explained in the mediator age was 29.0% (*p* < 0.001), for prohibited use, it was 20.9% (*p* < 0.001), dependence 21.6% (*p* < 0.001) and finance 8.5% (*p* = 0.036).

## Discussion

This research aimed to assess the strength of age as mediator between mobile phone use, psychopathological symptoms and gender on problematic mobile phone use and mobile phone addiction. Prohibited use and dependence had similar predictors regarding mobile phone use and activities, namely calls/day, time on the phone and using social media, suggesting there might be a considerable overlap between dependence and prohibited mobile phone use, i.e. when one is addressed, so might the other. Moreover, these phone use variables had both direct and indirect effects on prohibited use and dependence, suggesting the more an individual uses their phone for calls, texts and social media (i.e. for communication), the more prohibited and dependent their use can be. Importantly, the participants’ age was not responsible for time spent on the phone, calls made and social media, indicating risk for dependence and prohibited use could exist at any age. Older individuals in Generation X appear at risk for prohibited use and dependence, however not as much as younger individuals. This is contrary to previous literature (Jeong et al. 2016; Kwon et al. 2013) suggesting younger individuals (Generation Y) are more at risk for mobile phone use-related problems and addiction. For Generation X, the number of calls made predicts problematic and dependent use, whereas for Generation Y, this is predicted by time on the phone and social media use, suggesting although different age groups appear at risk for problematic mobile phone use, they engage in different usage patterns. Calling often in a day related directly to a higher prohibited use particularly for Generation X, suggesting there are different norms of behaviour associated with phone use in prohibited places for Generation Y and Generation X. These different behaviours may lead to problems, and therefore, communication styles may be important indicators of possibly problematic use differentiating between Generations X and Y. This suggests prevention efforts for problematic mobile phone use are needed for different age groups, but may be particularly beneficial for young populations where potentially problematic behaviours have not yet manifested. It is recommended to use qualitative research to assess the communication style differences between older and younger generations (i.e. synchronous vs asynchronous communication) to outline benefits and disadvantages of different forms of technology-mediated interaction and online/mobile community building and maintenance, particularly regarding differential effects on problematic mobile phone use.

Moreover, only for dependent (rather than prohibited) mobile phone use, stress appeared as significant predictor for both Generations X and Y, indicating stress may differentially impact upon problems experienced through mobile phone use. Some individuals may use their mobiles to cope with everyday stress, similar to individuals using the Internet to cope with and compensate for problems, potentially leading to addiction-related symptoms (Kardefelt-Winther 2014; Kuss et al. 2017). Accordingly, in the case of dependent use, mobile phone use can be considered a dysfunctional coping method which may offer a short-term solution to stressful experiences, but has potential damaging long-term effects. In the context of prevention and treatment efforts, focusing on developing functional coping mechanisms may reduce the reliance on dysfunctional mobile phone use-related coping.

Contrary to predictions, depression and anxiety were not found to be important predictors of problematic mobile phone use. The only significant effect found was an indirect effect of anxiety on age group, suggesting Generation Y may be more anxious than Generation X, possibly due to Generation Y being more likely to experience a fear of missing out (FOMO) (Przybylski et al. 2013), leading to increased pressure to use mobile phones and social media. From a developmental perspective, it appears that whilst in the process of psychosocial maturation, younger individuals may be confronted with more anxiety, which eventually evens out as they become older and more mature (Motti-Stefanidi 2015). Similar trends may hold true for depression-related symptoms, but unlike previous research (Babadi-Akashe et al. 2014; Murdock 2013), this study has not found depression related to problematic mobile phone use. In line with the diagnostic criteria for depression (American Psychiatric Association 2013), one could theorise that if individuals are feeling depressed, they may be more likely to withdraw from others, potentially reducing mobile phone use. It is suggested that future research assesses the interaction effects between specific mobile phone uses and both depressive and anxiety symptoms, as there may be inverse relationships with communicative phone use.

Financial problems through mobile phone use were predicted by social media use only; however, this effect disappeared when including the respective generations. An explanation for this finding is that intention of social media use may be different between Generation X and Generation Y; Generation Y use social media mainly for entertainment and networking at minimum cost, while Generation X use social media for marketing and customer management, considered more costly. Therefore, social media use-related financial problems are more likely to be observed in Generation X.

Regarding the mediating effects of age group, using social media and anxiety significantly predicted belonging to Generation Y, with calls per day predicted belonging to Generation X. This finding suggests Generation Y are more likely to use asynchronous communication inherent in popular social media, whereas Generation X engage more in synchronous communication. The results are important particularly given previous media and communication research. Boyd (2014) stressed the importance of online communication and building online spaces for US teenagers as social media has become an integral element of youth culture, offering alternative spaces for expression and connection. Turkle (2013) found young individuals appear particularly keen to connect with each other using technology, whilst simultaneously being alone in their physical environments, effectively leading to them being “alone together”. From a psychological perspective, Generation Y’s reliance on asynchronous social media-based communication may lead to a decrease in fear associated with participating in “real life” social interactions, but may increase the likelihood of problems if used to excess (Park et al. 2010).

Unlike previous research (Lopez-Fernandez et al. 2017), no direct or indirect effects of mobile phone use on associated problems based on participants’ gender were found, suggesting the females in the present sample do not experience more problems through mobile phone use relative to males. It could be speculated that specifically tapping into direct social interactions engaged in via mobile phones (rather than entertainment-focused activities) may yield a more pronounced effect on problems experienced as suggested by previous research and therefore, it is recommended to replicate this research by focusing on assessing interaction effects between female gender and specific social activities engaged in on phones as predictive of problematic mobile phone use.

Moreover, contrary to the outlined hypotheses and previous research (Lopez-Fernandez et al. 2017), the engagement in activities other than social media use, namely gaming and browsing, did not significantly predict problematic mobile phone use. It can be hypothesised that this may have resulted from a majority of female participants in this study, and previous research has shown the number of female gamers is still relatively low relative to males (Kuss and Griffiths 2012), and females are significantly less likely to present with gaming-related problems in psychotherapy contexts (Kuss and Griffiths 2015). Future research should investigate gender differences in mobile phone use habits and possible resulting problems using longitudinal and qualitative studies.

Limitations include the cross-sectional nature of this research, which does not provide indications of causality, the recruiting of a self-selected sample and the use of self-reports rather than objective and behavioural measures. Moreover, the PMPU scale appeared problematic both in terms of the dangerous subscale (which had very low reliability) and the finance subscale (for which no relationships with the predictor variables were found). It is suggested to reconsider the use of these subscales in future research and/or replace them with questions that tap into the use of more sophisticated present-day smartphones (rather than mobile phones as used in the early 2000s), and validate the revised measure. In addition to this, calls per day seemed to have a flooring effect, and this suggested most people tended to use their phones for fewer calls, whilst time on the phone was found to have a ceiling effect, which suggested that people spent more than 30 min on their phones per day on average. The respective questions can be formulated in such a way in future research as to take this into consideration. It is recommended to conduct qualitative research with users to specify the kinds of problems they experience as a result of everyday mobile phone use, which will enrich future research on the topic. Moreover, it is recommended to measure actual mobile phone engagement, using objective behavioural data, possibly using an experience-sampling method (Hofmann et al. 2012).

The present findings have implications for prevention and awareness-raising efforts of possibly problematic mobile phone use for educators, parents and individuals themselves, particularly including dependence and prohibited use. Specifically, dedicated interventions are encouraged to foster coping skills in mobile phone users to decrease dysfunctional coping through mobile use. Moreover, similar to the treatment of Internet use-related problems, clinical interventions need to be developed that pay particular attention to the individual’s usage habits and maladaptive coping behaviours, and take into consideration different modes of communication (i.e. synchronous vs asynchronous) as these have shown to differ across age groups. Future research efforts are encouraged to assess mobile phone usage differences across age and gender and how these may impact differently on resultant problems, using longitudinal, experience-sampling and qualitative research.
